# Establishment and Validation of the Detection of TERT Promoter Mutations by Human Gliomas U251 Cell Lines

**DOI:** 10.1155/2021/3271395

**Published:** 2021-06-01

**Authors:** Huili Bai, Shunjie Bai, Xiaosong Li, Yangli Zhang, Ying Li, Fang He, Wei Cheng

**Affiliations:** ^1^The Center for Clinical Molecular Medical Detection, The First Affiliated Hospital of Chongqing Medical University, 1 Friendship Road, Yuzhong District, Chongqing 400016, China; ^2^Department of Clinical Laboratory, The First Affiliated Hospital of Chongqing Medical University, 1 Friendship Road, Yuzhong District, Chongqing 400016, China; ^3^Department of pharmacy, The First Affiliated Hospital of Chongqing Medical University, 1 Friendship Road, Yuzhong District, Chongqing 400016, China

## Abstract

Gliomas are the most common type of primary brain tumor, yet the prognosis for glioma patients remains poor. Mutations in the promoter region of the telomerase reverse transcriptase gene (TERTp) are associated with diagnosis and poor prognosis in gliomas. Here, we developed a precise and rapid Sanger sequencing assay to screen or TERTp mutations. We established the Sanger sequencing approach for the detection of TERTp mutations based on human glioma cell lines U251 and assessed the analytical validation by determining the accuracy, sensitivity, precision, and specificity. In our study, we verified the accuracy of Sanger sequencing by the real-time polymerase chain reaction method. Our data showed that TERTp mutations were detected at an analytical sensitivity of 10% per mutant. The precision and specificity validation also showed the desired results. In total, 147 glioma patients were investigated for TERTp mutations, and of each patient, clinical data and molecular characteristics were analyzed. We found that anaplastic oligodendroglioma had the highest frequency of TERTp mutations (66.7%). No differences in TERTp mutation frequency were observed between frozen tissue specimens and formalin-fixed and paraffin-embedded tissue. TERTp mutations were associated with older patients (≥45 years), whereas isocitrate dehydrogenase (IDH) mutations were inclined to a younger age (<45 years), frontal location, and pathologic stage II-III patients. IDH mutations were significantly associated with O6-methylguanine-DNA methyltransferase (MGMT) methylation (*P* = 0.003) and lower Ki-67 protein expression (*P* = 0.011). Moreover, MGMT methylation was enriched in IDH-mutant/TERTp-mutant gliomas, and Ki-67 protein expression was the highest in the IDH-wild type/TERTp-mutant group. Taken together, the findings of this study indicate the establishment of a rapid, precise, and practical Sanger sequencing technique for TERTp mutations in gliomas that may show promising results in clinical applications.

## 1. Introduction

Gliomas are one of the most frequent types of primary malignant tumors in the central nervous system (CNS), accounting for 75% of all primary malignant brain tumors [1–4]. The highly invasive ability of these tumors hampers curative surgical resection and makes disease recurrence ineluctable [5]. Therefore, accurate diagnosis in glioma subgroups is of utmost importance for proper management of patients who suffer from this disease [6]. In a rapidly developing era, the diagnosis of gliomas is not only based on histopathology but also based on additional biomolecular makers, such as 1p/19q codeletion and isocitrate dehydrogenase (IDH)1/2 mutations [7, 8]. Nonetheless, the 5-year prognosis of gliomas patients is still poor [9]. Thus, it is essential to identify novel biomolecular makers involved in malignant transformation to improve diagnosis, prognosis, and treatment responses.

Recently, dysfunction of telomerase reverse transcriptase (TERT), the catalytic subunit of telomerase, has been demonstrated to involve in telomere maintenance and cell proliferation, contributing to human disease progression such as melanomas [10], non-small-cell lung cancer [11], bladder cancer [12], hepatocellular carcinomas [13], thyroid cancer [14], and brain tumors [15, 16]. In previous studies, the mutations in the promoter region of telomerase reverse transcriptase (TERTp) were associated with tumor biology [17]. Intriguingly, TERTp mutations affected survival and disease recurrence in bladder cancer [12]. Moreover, TERTp mutations play important roles in several health problems such as gliomas [18]. Based on the 2016 World Health Organization (WHO) classification of CNS tumors, TERTp mutations have been incorporated into glioma diagnosis [19]. Telomerase activity plays a pivotal role in tumorigenesis and facilitates the development of different brain tumors, including astrocytomas, primary glioblastomas (GBMs), and oligodendrogliomas [20, 21]. The two most frequent mutations (chr5: 1,295, 228 C>T and 1,295, 250 C>T, respectively), located at –124 bp and –146 bp, independently generate an identical 11 bp nucleotide sequence containing a consensus binding site for E-twenty-six (ETS) transcription factors and have been associated with increased mRNA expression or TERTp transcriptional activity [22]. Knockdown of human TERT inhibited cell proliferation and migration of gliomas in vivo [23]. Hence, detecting TERTp mutations in a clinical setting is critical.

Current techniques, such as Amplification Refractory Mutation System PCR (ARMS-PCR) and droplet digital PCR (ddPCR) for mutation detection, are limited in terms of screening unknown mutations. Furthermore, next-generation sequencing (NGS) often takes a while to run, analyzing data is time-consuming, and it can be costly and of high requirement. Sanger sequencing is a widely used technique to interrogate genes for small fragment mutations and acts as a gold standard for mutation detection. In this study, we aimed to develop a Sanger sequencing assay to achieve the precise and rapid detection of the TERTp mutations in gliomas and simultaneously assessed analytical validation and clinical utility.

## 2. Materials and Methods

### 2.1. Clinical Samples

Glioma samples were collected from 147 patients who underwent surgery at The Department of Neurosurgery, the First Affiliated Hospital of Chongqing Medical University (Chongqing, China), between June 2016 and April 2019. Among the tumors were 37 astrocytomas (DA), 15 anaplastic astrocytomas (AA), 24 anaplastic oligodendrogliomas (AO), 6 oligoastrocytomas (OA), 28 oligodendrogliomas (O), and 37 GBMs. Patients were diagnosed in accordance with pathological diagnosis. Clinicopathological data, including sex, age, tumor location, histologic type, pathologic stage, and Ki-67 protein expression, were retrospectively retrieved from respective institutional medical record systems. Formalin-fixed paraffin-embedded (FFPE) tumor tissues (47, 31.97%) and frozen tissues (100, 68.03%) of 147 glioma cases underwent molecular detection. Our study was approved by the ethics committee of the First Affiliated Hospital of Chongqing Medical University (Chongqing, China). Written informed consent (no. 2020-420), including molecular genetic testing and medical record reviews, was obtained from each patient or their next of kin. Data were anonymized prior to analysis to protect the patients' identify.

### 2.2. Cell Lines

Two human glioma cell lines, including U251 and OL, were kindly provided by the Chongqing Key Laboratory of Neurobiology (Chongqing Medical University). Cell lines were maintained in high-glucose Dulbecco's modified Eagle's medium (DMEM, HyClone, USA) supplemented with 10% fetal bovine serum (FBS, Gibco, USA) and 1% penicillin/streptomycin at 37°C in 5% CO_2_. Genomic DNA from both two cell lines was extracted for analytical validation.

### 2.3. DNA Extraction

For FFPE samples, six 5 *μ*m thick sections were cut from each selected block, followed by one section that was H&E-stained. The tumor area was marked on the control slide, and the tumor cell purity was more than 50%. DNA was extracted from the tumor-rich areas using a universal FFPE DNA extraction kit (Amoy Diagnostics Co. Ltd., Xiamen, China). Part of the DNA samples was isolated from frozen sections as previously described [24]. Genomic DNA was extracted from human glioma cell lines using a commercial kit (Tiangen Biotech Co., Ltd., Beijing, China). The DNA concentration was quantified using a Qubit 3.0 fluorometer (Invitrogen, Carlsbad, CA, USA).

### 2.4. Sequencing of TERTp and IDH1/2 Mutations

DNA (4 ng) was amplified by PCR using specific primers presented in Table 1. The M13F and M13R primers used are a pair of universal sequence-tagged primers, and M13 primers were used as sequencing primers (Table 1). The PCR reaction was performed using the following program: initial denaturation at 95°C for 10 min, followed by 30 cycles at 95°C for 30 sec, primer annealing at 62°C for 30 sec, and 72°C for 1 min, final extension took place at 72°C for 7 min. Amplification PCR and PCR sequencing were performed using the BigDye® Direct Cycle Sequencing Kit (Applied Biosystems, Foster City, CA, USA) according to the manufacturer's guidelines. The DNA sequencing products were purified using the BigDye® XTerminator™ Purifcation Kit (Applied Biosystems, Foster City, CA, USA), then resolved by capillary electrophoresis on a 3500DX Genetic Analyser (Applied Biosystems, Foster City, CA, USA). The entire experiment took approximately 4 hours.

### 2.5. RT-PCR (Real-Time Polymerase Chain Reaction) for TERTp Mutations

TERTp mutations were screened by RT-PCR. The PCR reagent and probe were from a commercial kit (Genetron Health Co. Ltd., Beijing, China). PCR was carried out in four stages: activation the Uracil-DNA Glycosylase (UNG) at 37°C for 3 min; incubation at 95°C for 3 min to activate DNA polymerase; enrichment of the mutant allele for 15 cycles at 95°C for 15 sec and 65°C for 45 sec; and TaqMan PCR for 25 cycles at 95°C for 15 sec and 60°C for 45 sec (fluorescence collection). Target genes and the internal control were labeled with FAM and ROX probes, respectively. Reactions were carried out in single-sealed tubes and detected using a 7500 Real-Time PCR instrument (Applied Biosystems, Foster City, CA, USA).

### 2.6. Methylation-Specific PCR (MSP)

The methylation status of the MGMT gene promoter was detected by extracting tumor DNA for bisulfite conversion using the EZ DNA methylation-Gold Kit (Zymo Research, Orange County, CA, USA). Two separate MSP reactions were performed, one using primers specific for methylated MGMT promoter sequences and the other using PCR primers specific for unmethylated MGMT promoter sequences (Table 1) [25].

### 2.7. Statistical Analysis

GraphPad Prism 5 (La Jolla, CA, USA) was used for all statistical analysis (the Pearson chi-square test or the 2-tailed Fisher's exact test). Statistical significance was indicated as *P* < 0.05. Sequence homogeneity was confirmed by comparison of the available sequences on the NCBI's BLAST (basic local alignment search tool) and Chromas software. Cycle threshold (Ct) values [26] were recorded, and corresponding *Δ*Ct values (Ct (TERT target gene assay)-Ct (internal control assay) were calculated. Analytical validation experiments were performed in triplicate.

## 3. Results

### 3.1. The Establishment of Sanger Sequencing-Based Methods to Detect TERTp Mutations

All samples were successfully analyzed by Sanger sequencing. The contiguous read length of the sequence product was approximately 233 bp, and no dimer structure and nonspecific amplifications were observed, which was in line with the primer design software database. Based on the sequence analysis by BLAST, the output sequence showed 100% homology and perfectly matched with the TERT gene promoter region (data not shown). The reportable range also included the wild-type, C228T, and C250T mutation status, and it was clearly visible and readable. Moreover, the sequencing peak was clear without noise. Figure S1 presents Sanger sequencing data for representative samples.

### 3.2. Analytical Validation of the TERTp Sequencing Assay

The analytical validation of the TERTp sequencing assay was assessed by determining the accuracy, sensitivity, precision, and specificity.

**Figure 1:**
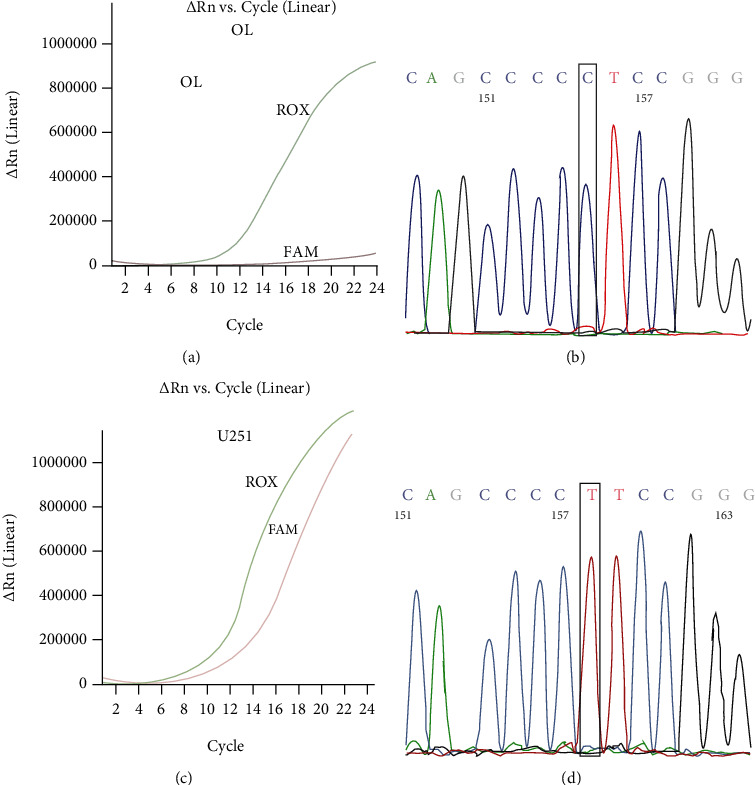
Accuracy of the Sanger sequencing assay for TERTp mutations in comparison with the RT-PCR method. Genomic DNA from OL cell lines was used for the detection of TERTp mutations by the RT-PCR method (a) and Sanger sequencing assays (b). OL cell lines presented wild-type TERTp. Genomic DNA from the U251 cell line was used for the detection of TERTp mutations by the RT-PCR method (c) and Sanger sequencing assays (d). U251 cell lines harbored C228T homozygous mutation of the TERTp. The TERTp target gene and internal control were labeled with FAM and ROX probes, respectively. TERTp: the promoter region of the telomerase reverse transcriptase gene.

For the analytical accuracy assay, 20 clinical samples were randomly investigated for TERTp mutations; 12 cases were wild-type, 7 cases contained a C228T mutation, and 1 case contained a C250T mutation. RT-PCR analysis confirmed the sequencing results (Table SI). To further verify the accuracy assay, we analyzed OL and U251 cell lines for TERTp mutations using RT-PCR assay and Sanger sequencing analysis. The results showed that the OL cell line was wild-type for TERTp (Figures 1(a) and 1(b)), whereas the U251 cell line harbored a TERTp C228T homozygous mutation (Figures 1(c) and 1(d)).

**Figure 2:**
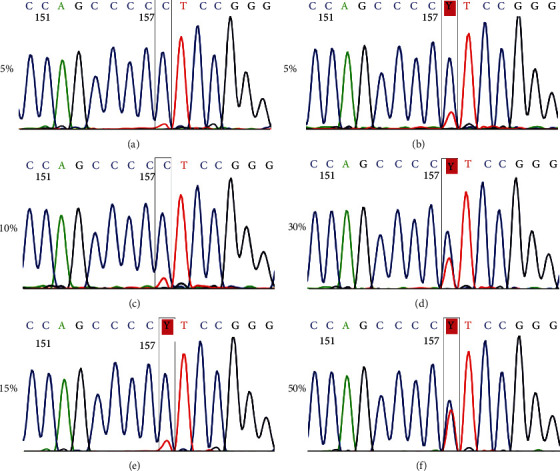
Sensitivity of the Sanger sequencing assay for the TERTp mutation. The percentage of C228T mutants in genomic DNA from mutant and wild-type cell lines (U251: C228T; OL: WT) is 5% (a), 10% (b), 15% (c), 20% (d), 30% (e), and 50% (f). TERTp: the promoter region of the telomerase reverse transcriptase gene.

For the analytical sensitivity assay, we tested the assay on serial dilutions of the C228T target mutation, which were generated by mixing definite ratios of DNA extracted from mutant and wild-type cell lines (U251: C228T; OL: WT) to a panel consisting of 5%, 10%, 15%, 20%, 30%, and 50% of mutants (Figures 2(a)–2(f)). Our data showed that our sequencing method can detect the TERT C228T mutation at a sensitivity of 10% or more (Figure 2(b)).

For the analytical precision assay, 50% and 15% proportion mutation samples were tested for 10 days, respectively (Table 2). The mean, standard deviation (SD), and coefficient of variation (CV) of the C228 T/C signal intensity ratio were calculated to assess analytical precision. In this study, the total, within-day, and between-day SD values were 0.12, 0.02, and 0.12 for 50% proportion mutation cases, respectively. The total, within-day, and between-day SD values were 0.93, 0.13, and 0.71 for 15% proportion mutation cases, respectively. The total, within-day, and between-day CV were 10.72%, 1.84%, and 10.00% for 50% proportion mutation cases, respectively. The total, within-day, and between-day CV were 13.02%, 2.26%, and 10.73% for 15% proportion mutation cases, respectively. Therefore, in the case of both high proportion (50%) and low proportion (15%) mutants, the CV did not exceed 15%, and we concluded that there was evidence of constant precision.

**Figure 3:**
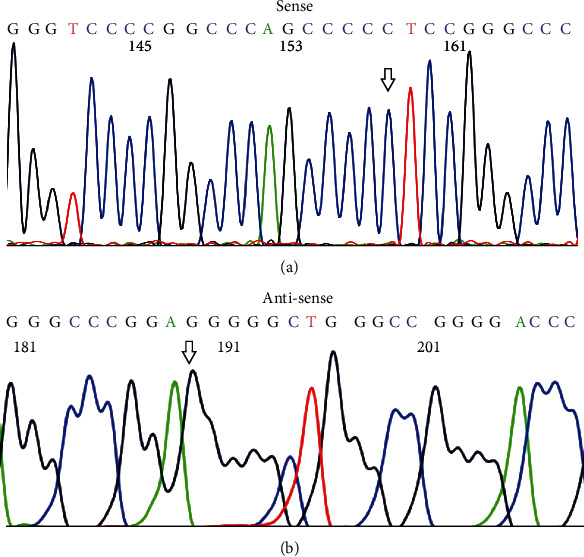
Specificity of the Sanger sequencing assay for TERTp mutations. Shown are representative sequences of wild-type TERTp. (a) Wild-type TERTp as detected by sense sequences. (b) Wild-type TERTp as detected by antisense sequences. Note that the arrowhead points to the nucleotide C228 site of TERTp. TERTp: the promoter region of the telomerase reverse transcriptase gene.

For the analytical specificity assay, the same sample was amplified in duplicate, one for TERTp forward sequencing (Figure 3(a)) and the other for TERTp reverse sequencing (Figure 3(b)). We obtained the same sequence results from forward and reverse sequencing and found a perfect match with the TERTp gene region.

### 3.3. TERTp Mutations Detected by Sanger Sequencing in Clinical Glioma Samples

**Figure 4:**
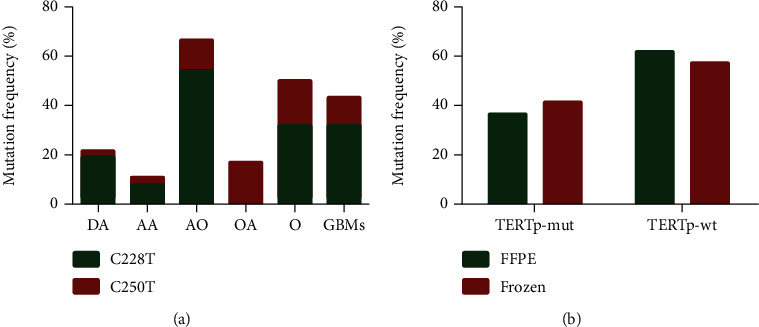
The frequency of TERTp mutations in glioma samples. (a) The frequency of TERTp C228T and C250T mutations in each glioma type (DA, AA, AO, OA, O, and GBMs). (b) The frequency of TERTp mutations in FFPE and frozen tissues. TERTp: the promoter region of the telomerase reverse transcriptase gene. DA: diffuse astrocytoma; AA: anaplastic astrocytoma; AO: anaplastic oligodendroglioma; OA: oligoastrocytoma; O: oligodendroglioma; GBMs: glioblastomas; FFEP: formalin fixed paraffin-embedded; TERTp-mut: TERT promoter mutation; TERTp-wt: TERT promoter wild type.

TERTp mutations were found in 59 out of 147 gliomas samples (40.13%). The C228T mutation (44 of 59) was much more frequent than the C250T mutation (15 of 59) in our cohort, and these mutations were mutually exclusive. The frequency of mutants for each histological type is presented in Figure 4(a). The incidence of TERTp mutations was the highest in AO tumor samples (66.7%), followed by DA (25.0%), AA (10.8%), and OA (16.7%) tumor samples showing a lower incidence, while O and GBM tumor samples (O, 56.5%; GBMs, 43.2%) showed an intermediate incidence when compared with others.

Comparative analyses were performed for the detection of TERTp mutations using a cohort of frozen tissue specimens (*n* = 100) and FFPE samples (*n* = 47). Representative H&E staining for FFPE and frozen tissues are shown in Figure S2. As shown in Figure 4(b), the median percentage of TERTp mutations from FFPE tissue and frozen tissue was 37.0% and 42.0%, respectively. No obvious differences were observed between the two tissue specimens.

### 3.4. Association of TERTp Mutations, IDH Mutations, and MGMT Methylation with Clinicopathological Features from Glioma Patients

Subsequently, we associated TERTp mutations, IDH mutations, and MGMT methylation with clinicopathological features from glioma patients and pooled analysis was performed, including sex, age, tumor location, histology, pathologic stage, and Ki-67 protein expression. Representative examples of the IDH status, Ki-67 staining, and MGMT status are shown in Figure S3 and S4. As shown in Table 3, TERTp mutations were common in glioma patients over 45 years old (*P* < 0.001). However, no associations were observed with tumor location, pathologic stage, and Ki-67 protein expression. As shown in Table 4, IDH mutations were highly associated with a younger age (<45 years, *P* = 0.005), frontal location (*P* < 0.001), and pathologic stage II–III (*P* < 0.001). IDH mutations were more frequent in MGMT methylated cases compared with unmethylated cases (*P* = 0.003). Furthermore, IDH mutations correlated with low Ki-67 protein expression (<15%, *P* = 0.011), and MGMT methylation was more frequently detected in frontally located gliomas (*P* = 0.022). It was independent of pathologic stage, TERTp mutations, and Ki-67 protein expression.

### 3.5. Association of Both TERTp Mutations and IDH Mutations with MGMT Methylation and Ki-67 Protein Expression

**Figure 5:**
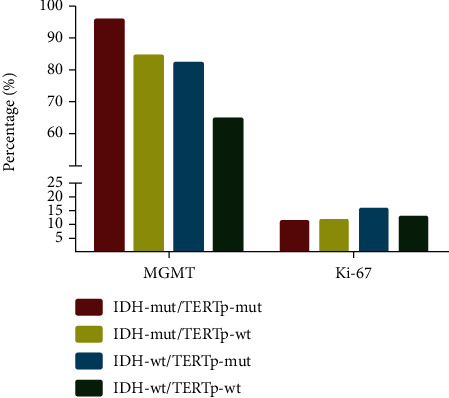
The percentage of MGMT methylation and Ki-67 protein expression in four gliomas subgroups. MGMT methylation was enriched in the IDH-mut/TERTp-mut subgroup, and the percentage of Ki-67 protein expression was the highest in the IDH-wt/TERTp-mut subgroup. MGMT: O6-methylguanine-DNA methyltransferase; TERTp: the promoter region of the telomerase reverse transcriptase gene; IDH: isocitrate dehydrogenase; mut: mutation; wt: wild-type.

Glioma cases were clustered into four groups: IDH-mut/TERTp-mut (positive for both IDH and TERTp mutations), IDH-mut/TERTp-wt (positive IDH mutation, but negative for TERTp mutations), IDH-wt/TERTp-mut (positive TERTp mutations, but negative for IDH mutations), and IDH-wt/TERTp-wt (no IDH or TERTp mutations). As shown in Figure 5, MGMT methylation was enriched in IDH-mut tumors, especially in the IDH-mut/TERTp-mut group.

In this study, we clinical information was collected to analyze the expression of Ki-67 protein, which is a maker of tumor proliferation [27]. Our data showed that Ki-67 protein expression was the highest in the IDH-wt/TERTp-mut group, followed by IDH-wt/TERTp-wt, which also showed high levels of Ki-67 protein expression.

## 4. Discussion

In this study, we developed and evaluated a Sanger sequencing assay of TERTp mutations. Our assay was more rapid than conventional sequencing and can be performed in less than 4 hours. Notably, our method is useful for the 10% of mutant tumor samples, in which high sensitivity mutation detection is needed for samples with very a low DNA input (4 ng). Clinical tumor tissues are rare and precious. In addition, the use of universal primers may simplify postamplification procedures by allowing all sequencing reactions to be performed with M13F and M13R primers [28]. In previous studies, it was shown that Sanger sequencing lacked the sensitivity for use on glioma samples, which could be prone to low tumor purity and heterogeneity of the gliomas themselves [28, 29]. To overcome these potential problems, all tumor tissue sections were reevaluated by a professional pathologist to obtain ≥50% higher-purity tumor areas. In some cases, other approaches for mutation detection may have specific advantages. For instance, NGS can simultaneously analyze many molecular alterations with high sensitivity. Other methods, including ARMS-PCR and ddPCR, are highly sensitive and convenient. Moreover, studies have demonstrated that the sensitivity of ARMS-PCR and ddPCR is 0.1% and 0.01%, respectively [30, 31]. However, whether such a high sensitivity method is required is still controversial as low proportion mutation might be not required for clinical applications.

We found that U251 cells harbored the C228T TERTp mutation and that OL cells did not carry C228T or C250T TERTp mutations. It is particularly important to identify the underlying mechanism for the malignant biological properties of gliomas in vitro study. Indeed, immortalized cell lines can provide strong evidence in the cancer setting [32]. We identified a novel C229T+C228T mutation in a single case of thyroid cancer (data not shown). In addition, the assay may have utility in other genetic mutations, such as IDH1/2 mutations in gliomas [33], and genetic makers can improve clinical targeted therapy development.

In the present study, we performed sequence analysis of TERTp mutations in a cohort of 147 gliomas tumor samples. The results of our study suggested that anaplastic oligodendrogliomas exhibited the highest incidence of TERTp mutations across all glioma types, followed by oligodendroglia. Data were in line with the findings presented in previously published studies [34, 35]. The strong association between TERTp mutations and the histology of oligodendroglial tumors suggests that TERTp mutations are involved in oncogenesis of oligodendroglial tumors [22]. However, in some studies, it was presented that the highest frequency of TERTp mutations was GBMs [22, 36]. The discrepancy may be explained by population differences, regional differences, or tumor sample size. Most reports on this subject are from Western populations; however, our present study was based on the Han Chinese population. Furthermore, we evaluated FFPE tissue samples and frozen tissue specimens for TERTp mutations. The frequency of FFPE specimens was slightly lower compared to frozen tissues regarding TERTp mutations (37.0% vs. 42.0%, respectively). This may be because the use of FFPE tissue samples could give rise to high false-negative rates, and a large proportion of normal tissue reduced the positive rate of mutant cancer cells [37]. In a recent study, it was revealed that formalin fixation might result in the deamination of cytosine residues, DNA degradation, and single-nucleotide variants [38, 39]. However, FFPE samples are suitable for long-term storage and can be widely used for molecular detection [40]. Therefore, our Sanger sequencing for TERTp mutations can be applied to routinely process frozen tissue or FFPE tissue and provided the required DNA quality and tumor purity.

We additionally investigated the relationship between three biomarkers (TERTp, IDH1/2, and MGMT methylation status) and clinicopathological features. In line with recent studies [41], the frequency of TERTp mutations correlated with an older age. TERTp mutations could accelerate cancer cell proliferation and DNA repair [42]. Taken together, these findings may explain the fact that TERTp mutations could be one of the main causes in elderly patients. On the contrary, IDH mutations were predominantly found in younger patients, at a frontal location, and in pathologic stage II-III patients, which agreed with prior studies [42, 43]. Moreover, in the context of our findings, IDH mutations were significantly associated with MGMT promoter methylation. Prior studies have shown that patients harboring IDH mutations have an improved outcome when compared with IDH wild-type patients [44]. Lu et al. demonstrated that IDH mutations changed the enzyme substrate affinity, leading to the methylation of cytosine-phosphate-guanine islands, including the MGMT promoter [45].

Ki-67 protein expression is associated with cell cycle and accelerates tumor progression by promoting cell proliferation and metastasis [46]. Our findings indicated that Ki-67 protein expression was lower in IDH mutation tumors and was the highest in the IDH-wt/TERTp-mut group, thereby indicating that IDH-wt and TERTp-mut have a greater capacity to promote tumor growth and progression. Data by Coons et al. supported the view that Ki-67 protein expression was related to the proliferative activity and outcome of oligodendrogliomas [47].

In our study, we successfully established a Sanger sequencing-based molecular screening method for detecting TERT promoter mutations in glioma samples. Compared to traditional sequencing, this method is more rapid, convenient, and simple. We found that glioma cell line U251 harbored the C228T TERTp homozygous mutation and helped us with performance verification. Moreover, we completed the association of both TERTp mutations and IDH mutations with MGMT methylation and Ki-67 protein expression. Because the study contains a limited number of samples, investigating the distribution frequency of the above-mentioned four groups in each histological type could not be performed. However, this lack of data will not affect a clinician's decisions about a patient's treatment. In summary, a simple and accurate method for screening of TERTp mutations may aid in and earlier diagnosis and prognosis evaluation of gliomas.

## Figures and Tables

**Table 1:** Primer sequences per gene.

Genes	Forward primer (5′-3′)	Reverse primer (5′-3′)	Amplified fragments
TERT	M13F-AGTGGATTCGCGGGCACAGA	M13R-CAGCGCTGCCTGAAACTC	235 bp
IDH1	M13F-GAGAAGAGGGTTGAGGAGTT	M13R-TACCTTGCTTAATGGGTGTA	294 bp
IDH2	M13F-GCTTGGGGTTCAAATTCTG	M13R-CGGTCTGCCACAAAGTCTG	343 bp
M13-tagged primer	TGTAAAACGACGGCCAGT	CAGGAAACAGCTATGACC	/
MSP-M	TTTCGACGTTCGTAGGTTTTCGC	GCACTCTTCCGAAAACGAAACG	81 bp
MSP-U	TTTGTGTTTTGATGTTTGTAGGTTTTTGT	AACTCCACACTCTTCCAAAAACAAAACA	93 bp

TERT: telomerase reverse transcriptase; IDH1: isocitrate dehydrogenase 1; IDH2: isocitrate dehydrogenase 2; M13F: M13 universal forward sequence-tagged primers; M13R: M13 universal reverse sequence-tagged primers. MSP-M: methylation special PCR methylated primers; MSP-U: methylation special PCR unmethylated primers.

**Table 2:** Precision of the Sanger sequencing assay for TERTp mutations. Performance of interassay and intra-assay coefficients of variation.

Concentration	*n*	Total			Within-day			Between-day		
		Mean	SD	CV (%)	Mean	SD	CV (%)	Mean	SD	CV (%)
50%	30	1.14	0.12	10.72	1.21	0.02	1.84	1.20	0.12	10.00
15%	30	7.14	0.93	13.02	5.67	0.13	2.26	6.61	0.71	10.73

TERTp: the promoter region of the telomerase reverse transcriptase gene; SD: standard deviation; CV: coefficient of variation.

**Table 3:** Correlations between clinicopathological characteristics and the status of TERTp mutations.

	Total	TERTp		*P* value
		Mut	Wt	
	*n* = 147 (%)	*n* = 59 (%)	*n* = 88 (%)	
Sex				0.041
Male	84 (57.1)	40 (67.8)	44 (50.0)	
Female	63 (42.9)	19 (32.2)	44 (50.0)	
Age (years)				<0.001^∗^
<45	70 (47.6)	17 (28.8)	53 (60.2)	
45-60	59 (40.1)	31 (52.5)	28 (31.8)	
≥60	18 (12.2)	11 (18.6)	7 (8.0)	
Tumor location				0.729
Frontal	91 (61.9)	38 (64.4)	53 (60.2)	
Others	56 (38.1)	21 (35.6)	35 (39.8)	
Histology				0.007^∗^
DA	37 (25.2)	8 (13.6)	29 (33.0)	
AA	15 (10.2)	4 (6.8)	11 (12.5)	
AO	24 (16.3)	16 (27.1)	8 (9.1)	
OA	6 (4.1)	1 (1.7)	5 (5.7)	
O	28 (19.0)	14 (23.7)	14 (15.9)	
GBMs	37 (25.2)	16 (27.1)	21 (23.9)	
Pathologic stage				0.338
Grade II-III	109 (74.1)	41 (69.5)	68 (77.3)	
Grade IV	38 (25.9)	18 (30.5)	20 (22.7)	
Ki-67				0.392
<15%	89 (60.5)	33 (55.9)	56 (63.6)	
≥15%	58 (39.5)	26 (44.1)	32 (36.4)	
IDH gene				0.312
Mutation	76 (51.7)	34 (57.6)	42 (47.7)	
Wild type	71 (48.3)	25 (42.4)	46 (52.3)	
MGMT promoter				0.088
Methylated	108 (73.5)	48 (81.4)	60 (68.2)	
Unmethylated	39 (26.5)	11 (18.6)	28 (31.8)	

TERTp: the promoter region of the telomerase reverse transcriptase gene; IDH: isocitrate dehydrogenase; MGMT: O6-methylguanine-DNA methyltransferase; Mut: mutation; Wt: wild-type; DA: diffuse astrocytoma; AA: anaplastic astrocytoma; AO: anaplastic oligodendroglioma; OA: oligoastrocytoma; O: oligodendroglioma; GBMs: glioblastomas. *P* value was calculated by the Pearson chi-square test or the 2-tailed Fisher's exact test. ^∗^Statistically significant: *P* < 0.05.

**Table 4:** Correlations between clinicopathological characteristics and the status of TERTp mutations.

	Total	IDH		*P* value	MGMT		*P* value
		Mut	Wt		*M*	*U*	
	*n* = 147 (%)	*n* = 76 (%)	*n* = 71 (%)		*n* = 108 (%)	*n* = 39 (%)	
Sex				0.505			0.707
Male	84 (57.1)	41 (53.9)	43 (60.6)		63 (58.3)	21 (53.8)	
Female	63 (42.9)	35 (46.1)	28 (39.4)		45 (41.7)	18 (46.2)	
Age (years)				0.005^∗^			0.257
<45	70 (47.6)	42 (55.3)	28 (39.4)		50 (46.3)	20 (51.3)	
45-60	59 (40.1)	31 (40.8)	28 (39.4)		47 (43.5)	12 (30.8)	
≥60	18 (12.2)	3 (3.9)	15 (21.1)		11 (10.2)	7 (17.9)	
Tumor location				<0.001^∗^			0.022^∗^
Frontal	91 (61.9)	58 (76.3)	33 (46.5)		73 (67.6)	18 (46.2)	
Others	56 (38.1)	18 (23.7)	38 (53.5)		35 (32.4)	21 (53.8)	
Histology				<0.001^∗^			0.009^∗^
DA	37 (25.2)	20 (26.3)	17 (23.9)		22 (20.4)	15 (38.5)	
AA	15 (10.2)	6 (7.9)	9 (12.7)		14 (13.0)	1 (2.6)	
AO	24 (16.3)	16 (21.1)	8 (11.3)		20 (18.5)	4 (10.3)	
OA	6 (4.1)	4 (5.3)	2 (2.8)		2 (1.9)	4 (10.3)	
O	28 (19.0)	22 (28.9)	6 (8.5)		24 (22.2)	4 (10.3)	
GBMs	37 (25.2)	7 (9.2)	29 (40.8)		26 (24.1)	11 (28.2)	
Pathologic stage				<0.001^∗^			0.286
Grade II-III	109 (74.1)	67 (88.2)	42 (59.2)		83 (76.1)	26 (66.7)	
Grade IV	38 (25.9)	9 (11.8)	29 (40.8)		25 (22.9)	13 (33.3)	
Ki-67				0.011^∗^			1.000
<15%	89 (60.5)	54 (71.1)	35 (49.3)		65 (60.2)	24 (61.5)	
≥15%	58 (39.5)	22 (28.9)	36 (50.7)		43 (39.8)	15 (38.5)	
MGMT promoter				0.003^∗^			—
Methylated	108 (73.5)	64 (84.2)	44 (62.0)		—	—	
Unmethylated	39 (26.5)	12 (15.8)	27 (38.0)		—	—	
TERT promoter				0.312			0.088
Mutation	59 (40.1)	34 (44.7)	25 (35.2)		48 (44.4)	11 (28.2)	
Wild type	88 (59.9)	42 (55.3)	46 (64.8)		60 (55.6)	28 (71.8)	

TERTp: the promoter region of the telomerase reverse transcriptase gene; IDH: isocitrate dehydrogenase; MGMT: O6-methylguanine-DNA methyltransferase; Mut: mutation; Wt: wild-type; M: methylated; U: unmethylated; DA: diffuse astrocytoma; AA: anaplastic astrocytoma; AO: anaplastic oligodendroglioma; OA: oligoastrocytoma; O: oligodendroglioma; GBMs: glioblastomas. *P* value was calculated by the Pearson chi-square test or the 2-tailed Fisher's exact test. ^∗^Statistically significant: *P* < 0.05.

## Data Availability

All data generated or analyzed during this study are included in this published article.

## Supplementary Materials

Supplementary Materials Figure S1: representative examples of TERTp mutations by Sanger sequencing in glioma patients. (A) wild-type. (B) TERTp C250T heterozygous mutation. (C) TERTp C228T heterozygous mutation. Figure S2: representative HE stains of frozen and FFPE tissues in glioma samples. (A) frozen tissues. (B) FFPE tissues. The HE stains images were taken at ×400 magnification. Scale bars represent 100 *μ*m. Figure S3: representative examples of IDH1 mutations by the Sanger sequencing and Ki-67 stains by immunohistochemistry. (A) IDH1 wild-type. (B) IDH1 R132H mutation (the nucleotide changes of G395A). (C) Ki-67 stains negative, Ki-67 < 15%. (D) Ki-67 stains positive, Ki-67 ≥ 15%. The Ki-67 stains images were taken at ×400 magnification. Scale bars represent 100 *μ*m. Figure S4: MSP of MGMT promoter status in glioma samples. Unmethylation samples showed only unmethylated band with no methylated MGMT, while methylated and unmethylated bands were found in MGMT methylation samples. MSP: Methylation-specific PCR; M: PCR product amplified by methylated-specific primers; U: PCR product amplified by unmethylated-specific primers; P: positive sample; N: negative sample; B: blank control. Table SI: comparison of TERTp mutations detected by the Sanger sequencing and RT-PCR method in glioma patients.
