# Supplementary material for: Establishment and Validation of the Detection of TERT Promoter Mutations by Human Gliomas U251 Cell Lines
Source: Biomed Res Int. 2021 Jun 1;2021:3271395. doi: 10.1155/2021/3271395 (PMC8187059; doi:10.1155/2021/3271395)
Supplement: Supplementary Materials — Figure S1: representative examples of TERTp mutations by Sanger sequencing in glioma patients. (A) wild-type. (B) TERTp C250T heterozygous mutation. (C) TERTp C228T heterozygous mutation. Figure S2: representative HE stains of frozen and FFPE tissues in glioma samples. (A) frozen tissues. (B) FFPE tissues. The HE stains images were taken at ×400 magnification. Scale bars represent 100 μm. Figure S3: representative examples of IDH1 mutations by the Sanger sequencing and Ki-67 stains by immunohistochemistry. (A) IDH1 wild-type. (B) IDH1 R132H mutation (the nucleotide changes of G395A). (C) Ki-67 stains negative, Ki-67 < 15%. (D) Ki-67 stains positive, Ki-67 ≥ 15%. The Ki-67 stains images were taken at ×400 magnification. Scale bars represent 100 μm. Figure S4: MSP of MGMT promoter status in glioma samples. Unmethylation samples showed only unmethylated band with no methylated MGMT, while methylated and unmethylated bands were found in MGMT methylation samples. MSP: Methylation-specific PCR; M: PCR product amplified by methylated-specific primers; U: PCR product amplified by unmethylated-specific primers; P: positive sample; N: negative sample; B: blank control. Table SI: comparison of TERTp mutations detected by the Sanger sequencing and RT-PCR method in glioma patients. [file 3271395.f1.docx]

**Supplementary material**


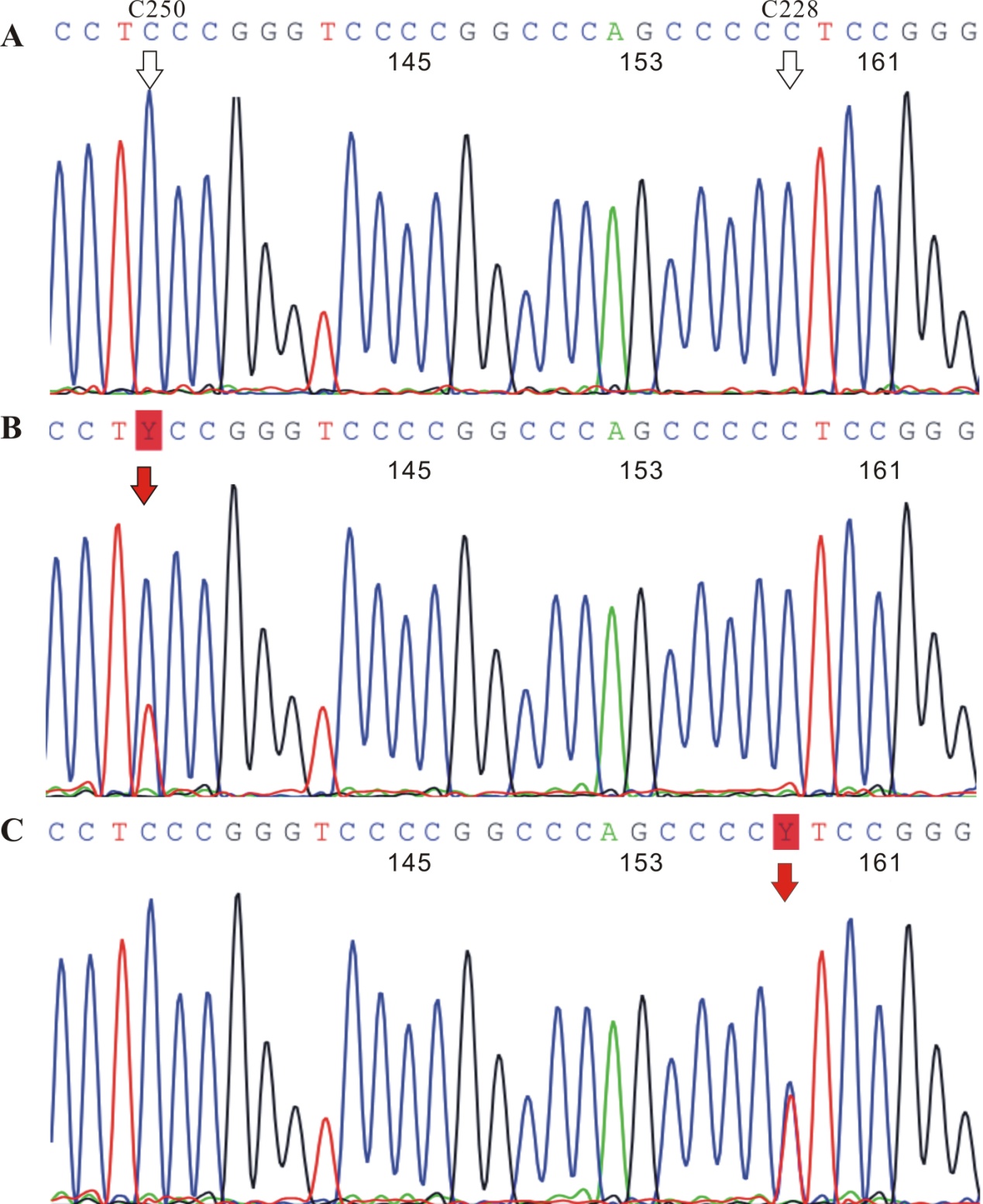


**Figure S1.** Representative examples of TERTp mutations by sanger sequencing in glioma patients. (A) wild-type. (B) TERTp C250T heterozygous mutation. (C) TERTp C228T heterozygous mutation.

**
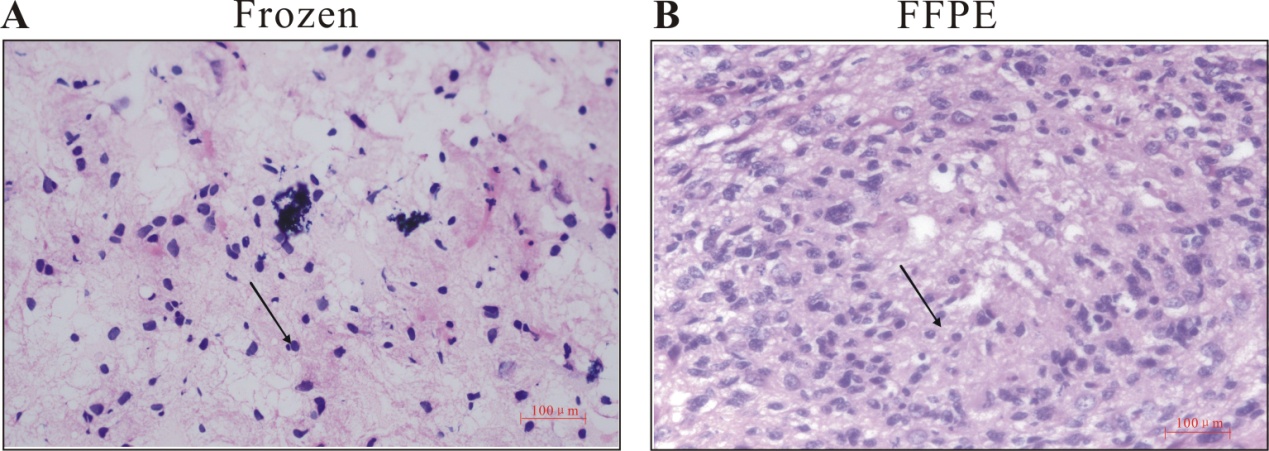
Figure S2.** Representative HE stains of frozen and FFPE tissues in glioma samples. (A) frozen tissues. (B) FFPE tissues. The HE stains images were taken at×400 magnification. Scale bars represent 100 μm.

**
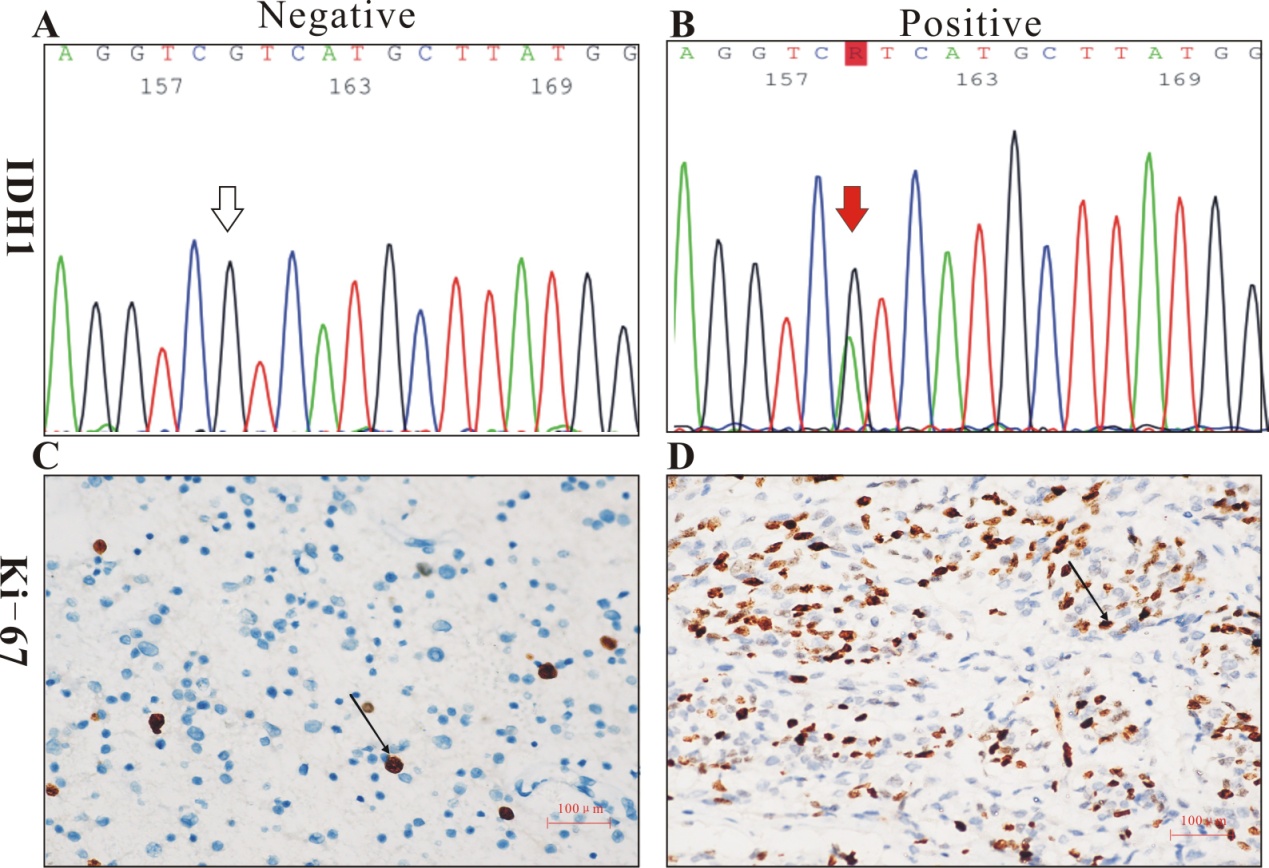
**

**Figure S3.** Representative examples of IDH1 mutations by the sanger sequencing and Ki-67 stains by immunohistochemistry. (A) IDH1 wild-type. (B) IDH1 R132H mutation (the nucleotide changes of G395A). (C) Ki-67 stains negative, Ki-67<15%. (D) Ki-67 stains positive, Ki-67≥15%. The Ki-67 stains images were taken at×400 magnification. Scale bars represent 100 μm.


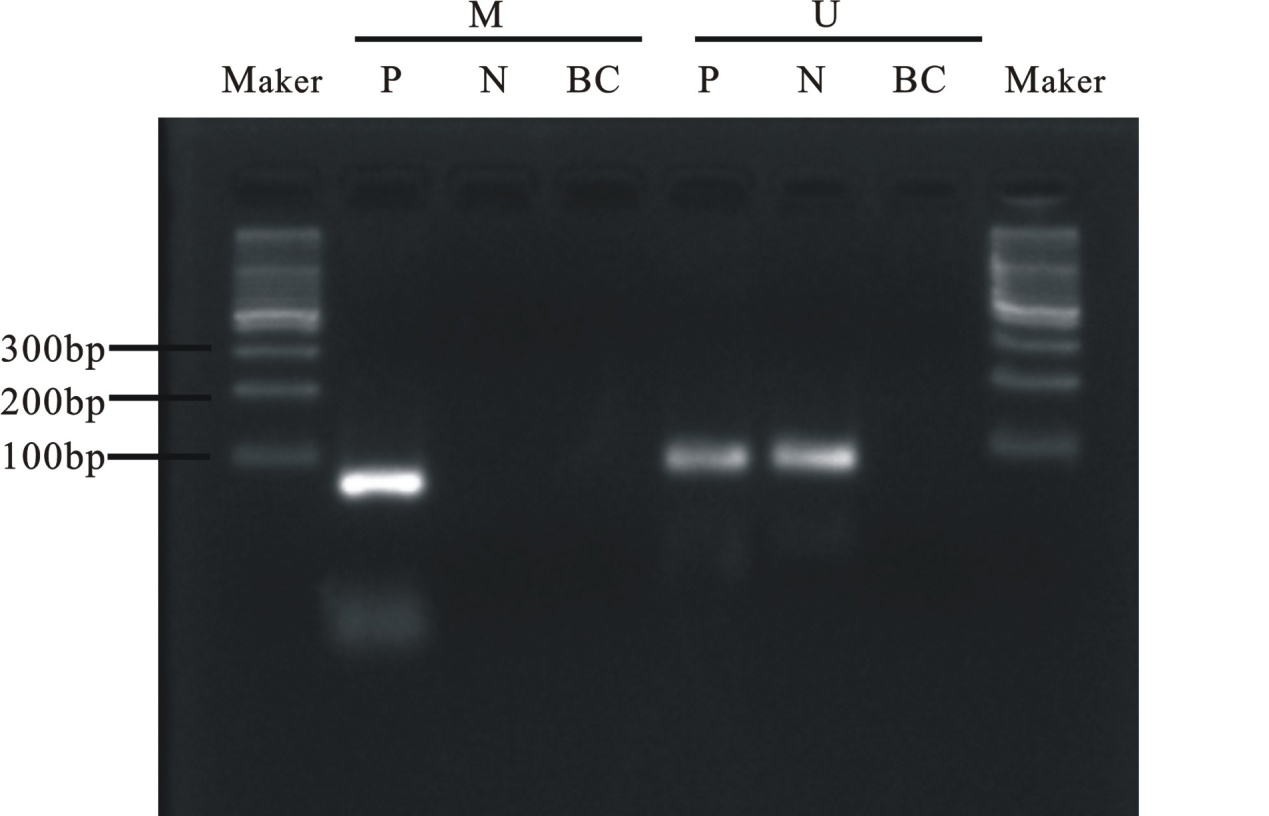


**Figure S4.** MSP of MGMT promoter status in glioma samples. Unmethylation samples showed only unmethylated band with no methylated MGMT, while methylated and unmethylated bands were found in MGMT methylation samples. Abbreviation: MSP, Methylation-specific PCR; M, PCR product amplified by methylated-specific primers; U, PCR product amplified by unmethylated-specific primers; P, positive sample; N, negative sample; B, blank control.

**Table SI**. Comparison of TERTp mutations detected by the sanger sequencing and RT-PCR method in glioma patients.

| Sanger sequencing | RT-PCR | | | Total | Concordance (%) | Agreement |
| --- | --- | --- | --- | --- | --- | --- |
|  | C228T | C250T | WT |  |  |  |
| C228T | 7 | 0 | 0 | 7 |  |  |
| C250T | 0 | 1 | 0 | 1 | 100.00 | 1.0 |
| WT | 0 | 0 | 12 | 12 |  |  |
| Total | 7 | 1 | 12 | 20 |  |  |

TERTp, the promoter region of the telomerase reverse transcriptase gene; RT-PCR, real-time polymerase chain reaction.
